# Gender, age, and concomitant diseases of melanosis coli in China: a multicenter study of 6,090 cases

**DOI:** 10.7717/peerj.4483

**Published:** 2018-03-08

**Authors:** Shufang Wang, Zikai Wang, Lihua Peng, Xiuli Zhang, Jianfeng Li, Yunsheng Yang, Bing Hu, Shoubin Ning, Bingyong Zhang, Junling Han, Ying Song, Gang Sun, Zhanguo Nie

**Affiliations:** 1 Department of Gastroenterology, Chinese PLA General Hospital, Beijing, China; 2 Department of Gasterology, West China Hospital, Chengdu, China; 3 Department of Gasterology, Air Force General Hospital, Beijing, China; 4 Department of Gasterology, Henan Provincial People’s Hospital, Zhengzhou, China; 5 Department of Gastrointestinal Endocrinology, 187 Military Hospital, Haikou, China; 6 Department of Gasterology, Xian Central Hospital, Xian, China; 7 Departmenf of Gasterology, Hainan Branch of the Chinese PLA General Hospital, Sanya, China; 8 Department of Gasterology, General Hospital of the Xinjiang Military Region, Urumqi, China

**Keywords:** Colonoscopy, Melanosis coli, Detection rate, Multicenter retrospective study, Epidemiology, Gastroenterology

## Abstract

**Backgrounds and Aims:**

Melanosis coli (MC) is a noninflammatory, benign, and reversible colonic disorder, but its detection rates in China are unclear. We therefore aimed to analyze the epidemiological characteristics of MC in China.

**Methods:**

We assessed the detection rates, associated factors and concomitant diseases of MC in the patients who underwent colonoscopy at eight medical centers across five regions of China between January 2006 and October 2016. All data were procured from the electronic database established at each participating institutions.

**Results:**

Among the 342,922 included cases, MC was detected in 6,090 cases (detection rate = 1.78%, 95% confidence interval, 1.73%–1.82%) at a mean age of 60 years. The detection rate gradually increased yearly, and along with the increasing age regardless of gender, while a rapid increase presented in the patients ≥60 years of age (0.58% for ≤25 years, 1.22% for 25–59 years, and 3.19% for ≥60 years). The detection rate was higher in females than in males; however, the rate of per-year increase was higher in males than in females at age of ≥60 years, which was 1.85-fold of that in females. Among cancer, polyp, inflammation, and diverticula, polyp was the most common concomitant disease of MC and identified in 41.72% of MC patients.

**Conclusions:**

MC detection rates were increased annually and elevated in older patients, particularly in male patients. Males in the elderly population of ≥60 years were most likely to have MC. Colonic polyp is the most common concomitant disease of MC.

## Introduction

Melanosis coli (MC) is a rare noninflammatory, benign, and reversible colonic disorder, pathologically manifested as excessive deposits of lipofuscin-like substances in the macrophages located in the lamina propria of colonic mucosa or rarely in the colonic submucosa, but in severe condition present in the extracellular component of the lamina propria (Debray et al., 1967; Dukes, 1935; Li, Browne & Ladabaum, 2009). In 1825, MC was initially reported and described as melanin pigmentation of the colonic mucosa by Billiard, while named as MC by Virchow in 1857 (Wittoesch, Jackman & Mc, 1958). Recently, the detection rate of MC has been incrementally increased along with the aging of the population, an increasing prevalence of constipation, and advances in colonoscopic diagnosis year by year (Shim & Lee, 2010). MC is more common in females than in males, with gradually younger onset age (Shim & Lee, 2010). Therefore, characterization of the onset age, gender, and increase in the MC’s incidence and detection rate will benefit the prevention and diagnosis of MC in clinical practice.

The pathological pigmentation in MC was initially considered to be the artificial outcome caused by staining of melanin with Masson Fontana reagents. Later, the staining was identified as a cross-reaction, and the pigment was recognized as lipofuscin, which could be stained with periodic acid–Schiff and long Ziehl–Neelsen methods (Ghadially & Parry, 1966; Steer & Colin-Jones, 1975). Many studies have demonstrated that this pigment is generated due to apoptosis of the colonic epithelial cells. Although the prevalence of MC has been increased in the past years and concurred with a variety colon diseases and conditions, except constipation and long-term laxative use (reversible after drug withdraw) as causing factors, the crelationship between MC and multiple colonic diseases remains uncertain and even overestimated in some cases (Biernacka-Wawrzonek et al., 2017; Byers et al., 1997; Coyne, 2013). However, the association of MC with colonic epithelial tumors has attracted substantial attention in the research field. The incidence probability of colorectal adenocarcinoma was elevated in the patients with MC according to some studies (Blackett et al., 2016). It remains unclear whether the causal relationship of MC with colorectal adenocarcinoma is invalid or because the colonic micropolyp is prone to be detected on the setting of brown or black mucosa. However, present epidemiological studies of MC in China were mostly single-centered with small numbers of subjects (Liu et al., 2017), which reflects neither the total incidence of MC in China nor the features of disease distribution and concomitant diseases.

The present study retrospectively analyzed the clinical data of 6,090 patients with MC from a huge sum of population of 342,922 subjects, who had underwent colonoscopy between January 2006 and October 2016 at eight medical centers located in five regions of China. The study aimed to examine the characteristics of disease distribution, gender, and age of MC, in addition to the concomitant diseases.

## Study Subjects and Methods

### Study subjects

This study was approved by the ethical review by Hainan Branch, General Hospital of PLA (301hnll-2017-05). Medical records of 342,922 patients who had undergone colonoscopy between January 2006 and October 2016 were reviewed including eight participating medical centers in China (Department of Gastroenterology, Chinese PLA General Hospital, Beijing, China; Department of Gastroenterology, West China Hospital, Chengdu, China; Department of Gastroenterology, General Hospital of Air Force, Beijing, China; Department of Gastroenterology, Henan Province People’s Hospital, Zhengzhou, China; Digestive and Endocrine Department, PLA187 Central Hospital, Haikou, China; Department of Gastroenterology, Xi’an Central Hospital, Xi’an, China; Department of Gastroenterology, Chinese PLA General Hospital Hainan Branch, Sanya, China; and Department of Gastroenterology, General Hospital of Xinjiang Military Region, Urumqi, China) located in five regions of China, namely Northern region, Central region, Southern region, Southwestern region, and Northwestern region. The clinical data of the study subjects with MC diagnosed by endoscopy were retrieved from the established endoscopy electronic databases of each gastrointestinal endoscopy center.

Subjects underwent colonoscopy for a variety of indications were grouped into four major areas: (1) IBD surveillance and status evaluation, (2) asymptomatic patients for cancer screening, (3) surveillance colonoscopy for a prior history of polyp or malignance, and (4) symptoms based examination.

The inclusion criteria were as follows: the patients who underwent colonoscopy or colonoscopic treatment as documented in the graphic system of gastrointestinal endoscopy center. The exclusion criteria were as follows: the patients in whom colonoscopy failed to reach the ileocecal junction defined as an incomplete colonoscopy, due to the inadequate preparation, intolerant pain, tortuosity, stricture, and intro-operative complications.

The diagnostic criteria of MC based on colonoscopy manifestation were as follows: the colonic mucosa looked smooth in brown, tan, or dark brown color, with the discoloration slight or very pronounced, and with changes in tiger stripe pattern, patch, or reticular cord, or with the appearance of areca section and the whole colonic lumen darker. Pathological evaluation was performed in the cases accompanied with other benign and malignant diseases on colonoscopy.

All of the data were retrieved for near 350,000 electronic records from the 8 medical centers in which the original data were saved in different format. A software engineer (Enming Zhou, Chongqing University, China) wrote a customized software program to procure these virtual data and depicting the readable figures and summarizing tables.

### Statistical analysis

All procured data were analyzed using the statistical software package of SPSS 13.0 (SPSS, Inc., Chicago, IL, USA). The categorical variables were expressed as a percentage.

## Results

### MC detection rate

**Figure 1:**
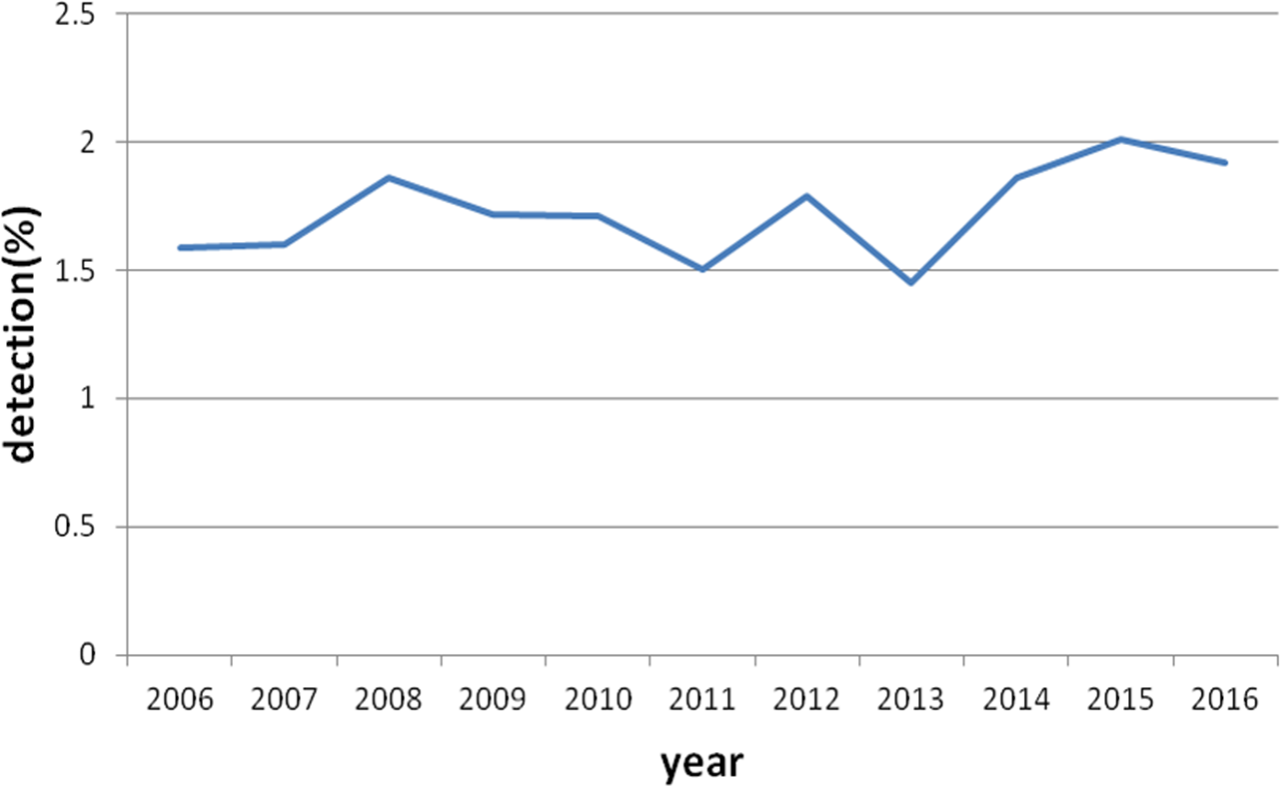
MC detection rate from year 2006 to 2016 in five regions of China. The MC detection rate curve of the last 10 years were shown a gradual rising trend in China.

Of the 342,922 patients who underwent colonoscopy, MC was detected in 6,090 cases (overall detection rate = 1.78%, 95% confidence interval, 1.73%–1.82%) comprising 2,646 males (0.77%, gender-specific detection rate 1.41%, 95% confidence interval, 1.36%–1.49%, 2,646/187,150) and 3,444 females (1.0%, gender-specific detection rate 2.21%, 95% confidence interval, 2.17%–2.24%, 3,444/155,772), with the mean age of 60 years (11–92 years). The ratio of male versus female patients was less than 1. The MC detection rates of the last 10 years were analyzed (Fig. 1), and the graph of the yearly MC detection rate displayed a gradual rising trend (*P* < 0.01).

**Table 1:** Relationship of age (WHO standard) with the overall detection rate of MC.

Age (year)	Total case	Case with MC	Case without MC	Detection rate (%)
≤44	115,862	992	114,870	0.85
45–59	126,492	1,887	124,605	1.49
60–74	82,699	2,094	80,605	2.53
75–89	17,700	1,110	16,590	6.27
≥90	169	7	162	4.14

**Notes.**

MC Melanosis coli WHO World Health Organization

### Characteristics of MC at different ages

**Figure 2:**
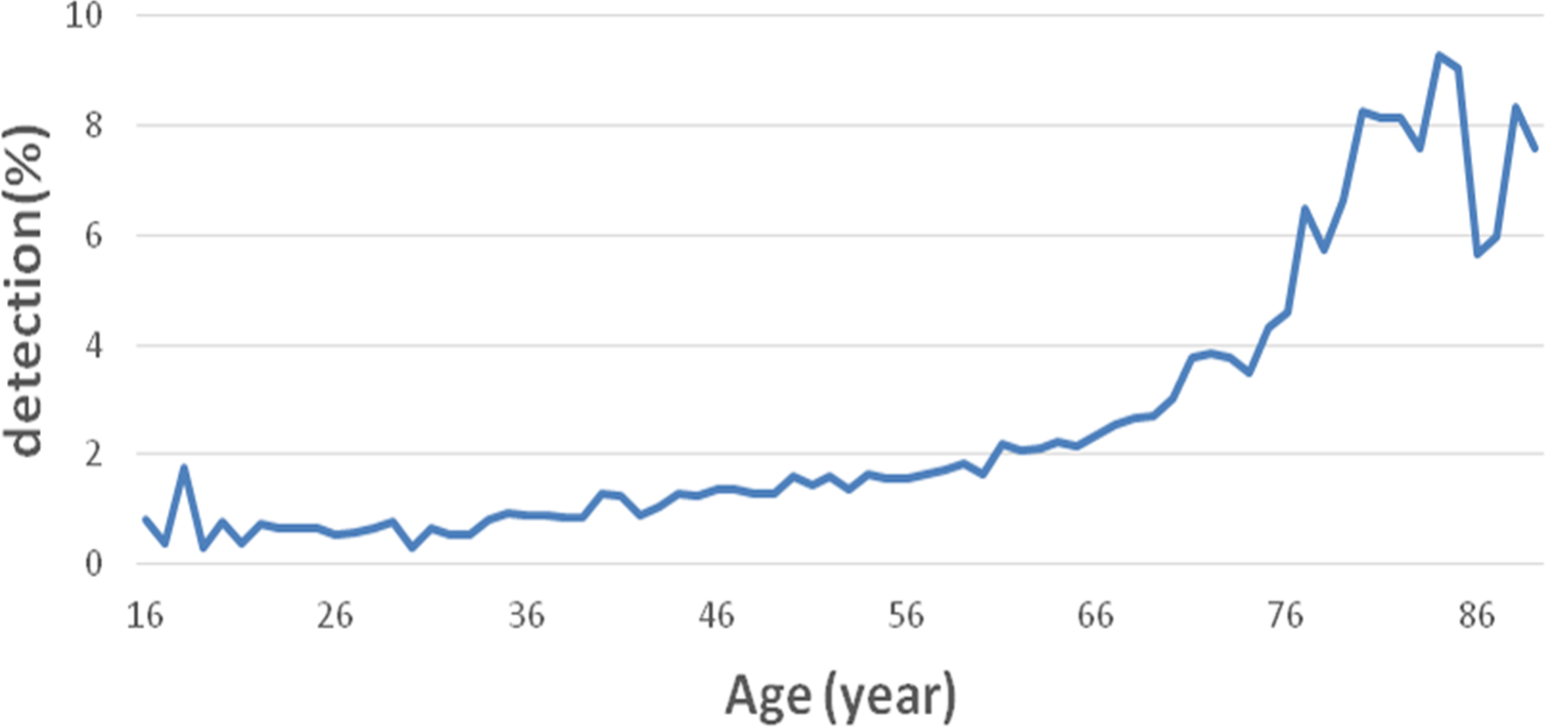
Overall detection rate of MC in different age groups. The MC incidence rate gradually increased with increasing age, and such increase initially slow, then increased rapidly in cases of after 60 years.

According to the World Health Organization (WHO) standard for age stratification, the five age groups of ≤44, 45–59, 60–74, 75–89, and ≥90 years were used. The MC detection rates in each group are listed in Table 1. In the group ≥90 years, the detection rate was 4.14% (7/169), which accounted for only 0.04% (169/6,090) of all subjects and therefore omitted for analyses. When the age stratification was simplified into three groups, <25, 25–59, and ≥60 years, the MC detection rates presented an increasing trend with increasing age, demonstrating a significant increase in patients older than 60 years of age, at 3.19% (3,211/100,568, *P* < 0.01) (Table 2). These results indicated that MC detection rate gradually increased with increasing age (Fig. 2). The increase was initially slow, which then increased rapidly after 60 years (Fig. 2).

**Table 2:** Relationship of age (simplified stratification) with the overall detection rate of MC.

Age (year)	Total case	Case with MC	Case without MC	Detection rate (%)
<25	13,296	78	13,218	0.58
25–59	229,058	2,801	226,257	1.22
≥60	100,568	3,211	97,357	3.19

**Notes.**

MC Melanosis coli

### Gender-specific increase in MC detection rate

**Figure 3:**
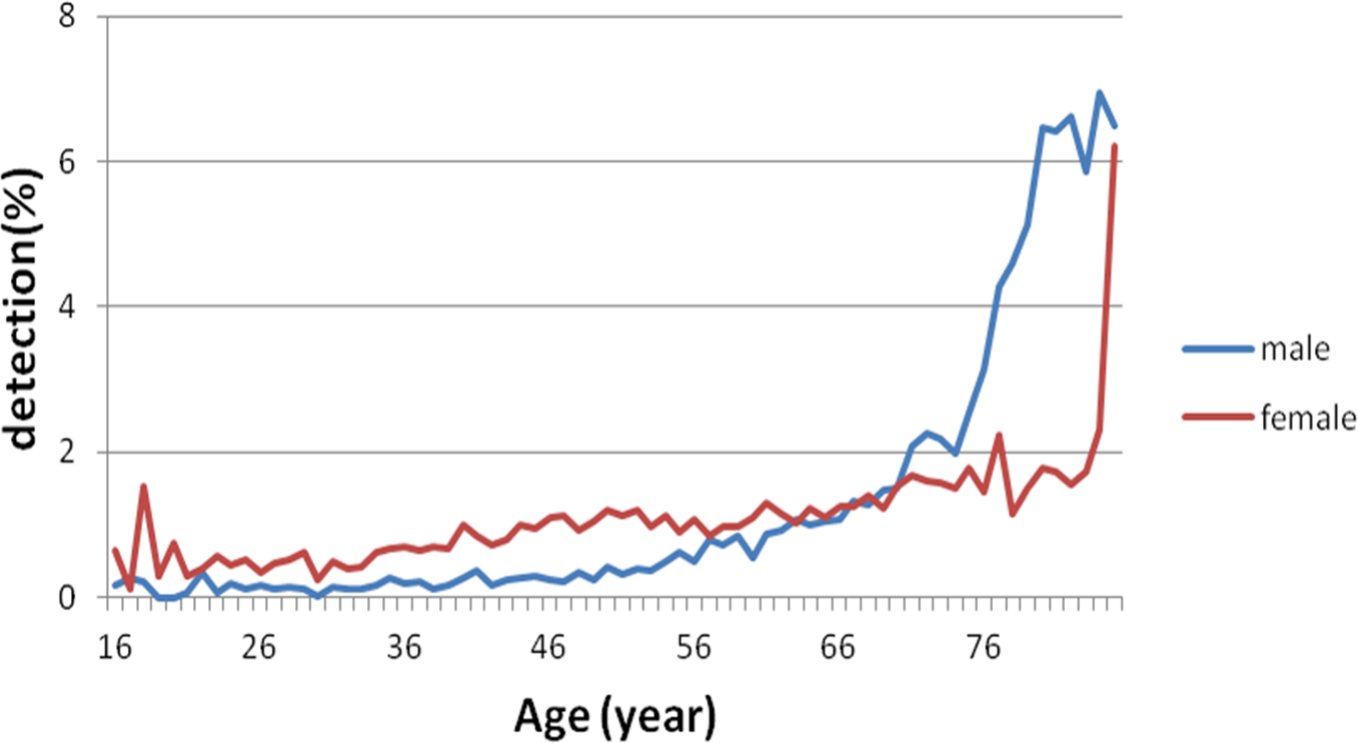
Gender-specific difference in the MC detection rate. For both male and female subjects, MC incidence gradually increased with increasing age.

**Figure 4:**
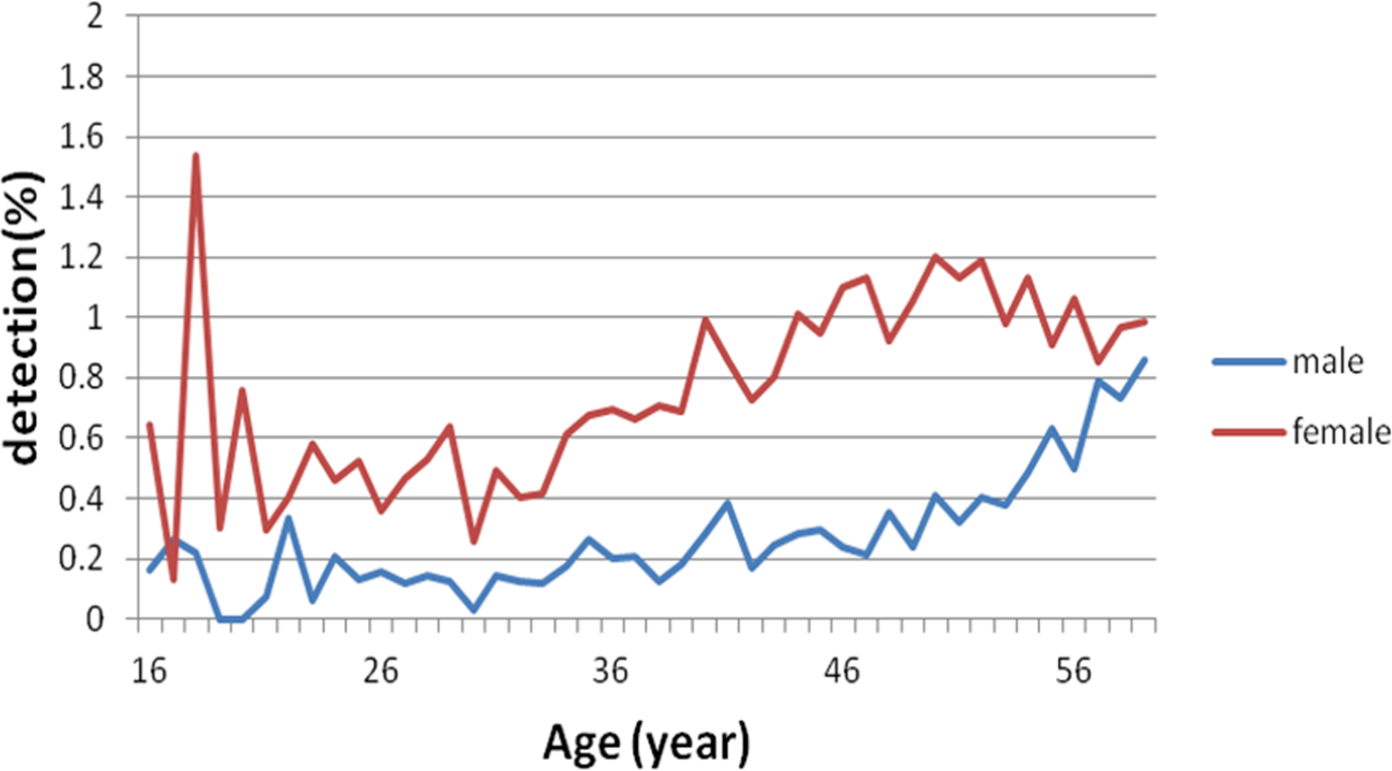
Gender-specific difference in the MC detection rate at age less than 60 years. The MC incidence rate was higher in females than in males below age of 60 years.

**Figure 5:**
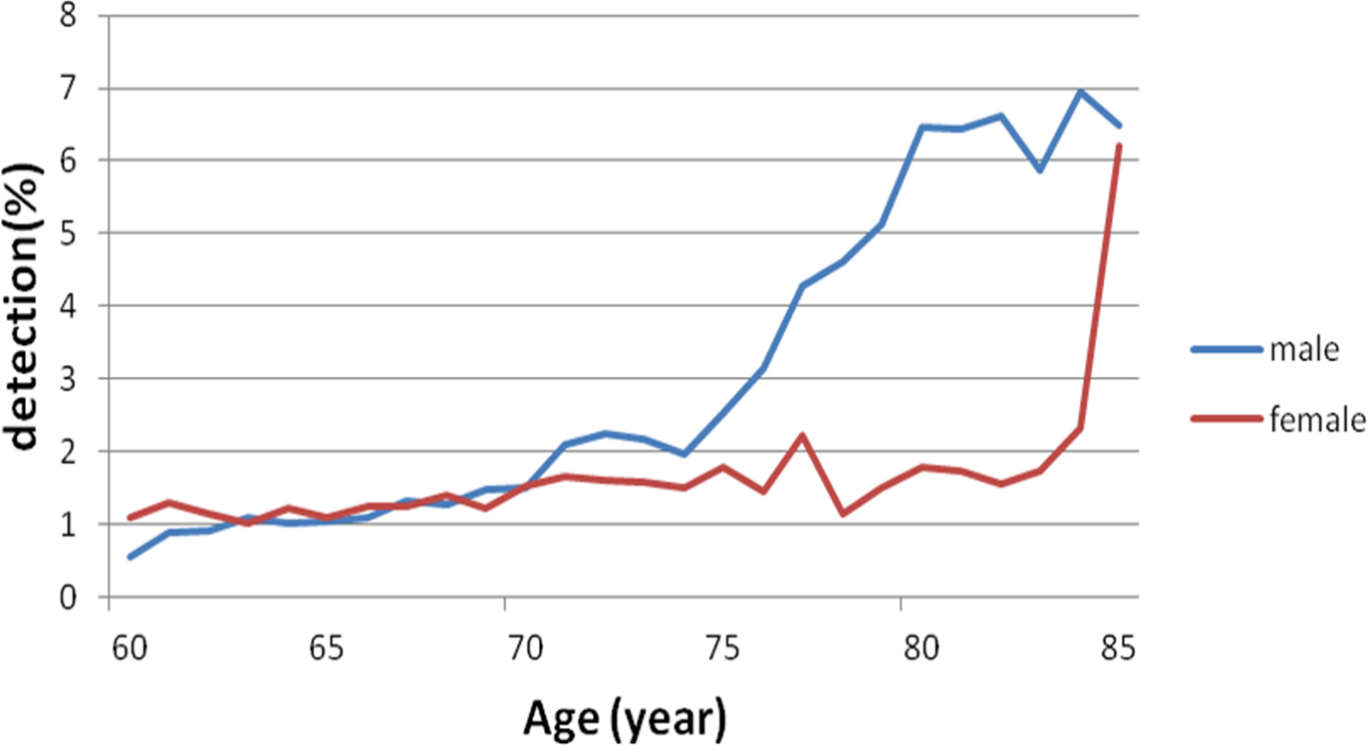
Gender-specific difference in the MC detection rate at age more than 60 years. The incidence increase rate presented an upward trend for both genders at age more than 60 years, while the increase was more rapid in males than in females, which was 1.85-fold of that in females. The increase rate in males surpassed that in females at age 75 years.

Regardless of gender, MC detection rate gradually increased with increasing age (Fig. 3). The MC detection rate was higher in females than in males below age of 60 years (*P* < 0.01, Fig. 4). In contrast, the detection rate increase rates were presented an increasing trend for both genders at age more than 60 years, while the increase was more prominent in males than in females, which was 1.85-fold of that in females (*P* < 0.01, Fig. 5). Thus, the increase in rate in males surpassed that in females at age 75 years (Fig. 5).

### Characteristics of MC concomitant diseases

**Figure 6:**
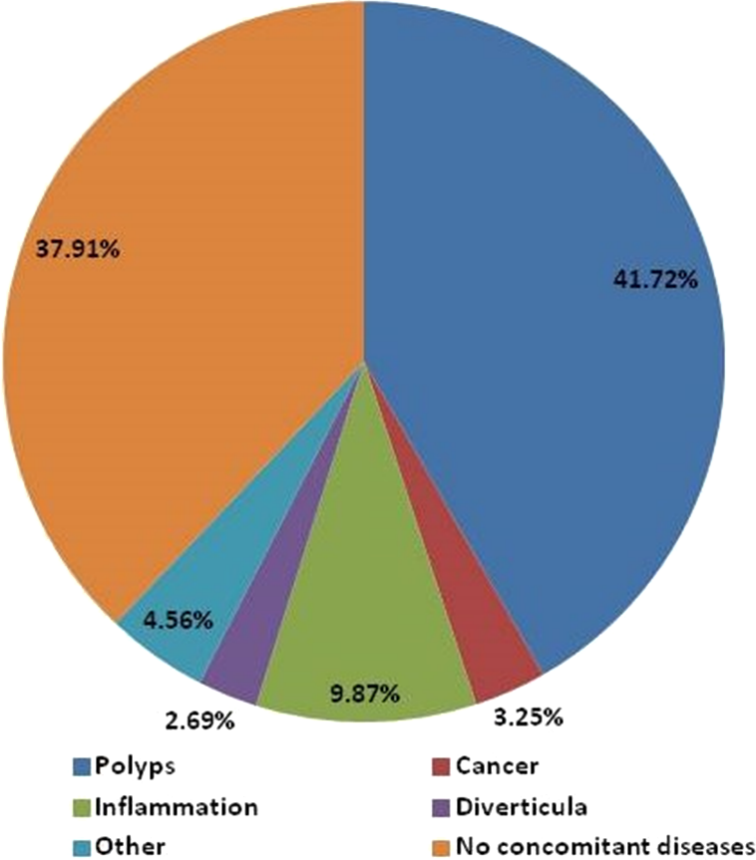
Characteristics of MC concomitant diseases. Colonic polyp is the most common concomitant disease of MC, accounting for 41.72%, followed by inflammation/colitis (9.87%), colorectal cancer (3.25%), and colonic diverticula (2.69%). Non-concomitant disease: MC, without concomitant diseases.

Colonic polyp is the most common concomitant disease of MC, accounting for 41.72% of all MC subjects, in which 33.6% was pathologically-confirmed adenomatous polyp. Other concomitant diseases included inflammation/colitis (9.87%), colorectal cancer (3.25%), and colonic diverticula (2.69%), detailed in Fig. 6. Compared to non-MC patients, the detection rates were higher in the following concomitant diseases: polyp (41.72% vs. 39.6%, *P* < 0.01), inflammation (9.87% vs. 8.52%, *P* < 0.01), cancer (3.25% % vs. 1.37%, *P* < 0.01), and diverticula (2.69% vs. 2.30%, *P* < 0.01).

## Discussion

This descriptive analysis reports the detection rate and associate factors of MC among the 350,000 subjects who had colonoscopy examination in the last 10 years at eight medical centers distributed across five regions of China. A retrospective analysis was also conducted on the 6,090 cases with a diagnosis of MC. This was to our knowledge a study with the widest geographic distribution and with the largest number of MC cases in China, which reflected the epidemiological characteristics of MC in China to a certain level.

In China, the MC incidence has presented an increasing trend with an aging population and the changes in diet composition and living environment. The detection rate in our study has exhibited a yearly rising trend, with a younger onset age as reported before (Shim & Lee, 2010). The MC incidence rate is around 10% or higher in Western nations (Thompson & Heaton, 1980), whereas the reported detection rate in China is significantly lower (1.78% in this study). The detection rate in this study is basically consistent with the references in China but lower than that in the Western population (Liu et al., 2017). It could be related to the ethnic differences, regional factors, dietary composition, consultation time, and clinical competence of endoscopy practitioners.

We found an association of age with MC detection rate in a Chinese population. Genetic analysis of MC colon tissues revealed a markedly reduced expression of P450 enzymes (CYP3A4, CYP3A7) and of glucuronosyltransferase family members (UGT2B11, UGT2B15) (Li et al., 2015), indicating the close association of metabolism with MC. In the present study, MC detection rate was higher in the middle-aged and elderly subjects than in the younger subjects, which was in accordance with previous reports of higher detection rate in the populations of no less than 60 years (Pellino et al., 2013). High MC detection rate in these populations could be attributable to alleviated metabolism. Aging is an important contributor to cell apoptosis, leading to evidently weakened capability of toxin metabolism, which can indirectly increase MC detection rate in middle-aged and elderly populations considering their slower metabolism (Devons, 2002). Middle-aged and elderly people are prone to constipation (Shim & Lee, 2010), which causes the prolonged duration of fecal retention in the colon and thus accelerates the absorption of toxins by colonic mucosa and stimulates hyperplasia, giving rise to an increased detection rate of colonic polyps and neoplasm. These lines of evidence might partially explain why the detection rates of MC concomitant diseases are higher than those of non-MC patient due to the aging-related pathogenesis of these benign and malignant disorders, such as polyp, inflammation, cancer, and diverticula. Chinese medicines such as anthraquinones, containing rhubarb, senna, and aloe, regularly taken by these populations have led to a significant increase in MC incidence (Benavides et al., 1997; Chen et al., 2011; Nusko et al., 1993). A previous study reported that 20% of people in developed countries take anthraquinones (Nusko et al., 1993), which promoted MC progression in some studies (Benavides et al., 1997; Chen et al., 2011; Nusko et al., 2000), leading to colonic epithelial cell apoptosis (Walker, Bennett & Axelsen, 1988). In addition, activity study of synthesized anthraquinones demonstrated that some of the active components in anthraquinones had potential genotoxic and carcinogenic properties (Mengs & Rudolph, 1993), making them an important factor inMC progression. Herbal tea was reported to induce the occurrence of MC (Kefeli et al., 2015). Regular wellness drink was also reported to cause MC (Kunkel et al., 2009). Therefore, propagation of MC information in populations with constipation can greatly benefit the prevention and treatment of MC, in addition to improvement in diet structure and medications of proper laxative.

Gender is closely related to MC. The MC detection rate is much higher in females than in males, and the difference can be associated with endocrine hormones in women (Coyne, 2013; Yang, Liu & Wang, 2010). In the present study, the MC detection rate was significantly higher in females than in males, consistent with previous reports. High detection rate of MC in females could be related to anti-obesity drugs reported to promote MC (Furukawa et al., 2011). In our study, we found the increase rate of MC detection rate was more rapid in elder male of ≥60 years than that of females. The real reasons still need to be explored. We supposed that might be associated with the decreased endocrine hormones in female.

In the present study, colonic polyp was the most common concomitant disease of MC. Previous studies demonstrated the close association between these diseases (Coyne, 2013; Nusko et al., 2000). It could be explained that the colonic mucosa was speckled with brownish pigmentation while colonic polyps appeared as pigment deficiency, which increased the detection rate of colonic polyps. Whether MC increases the risk for colon neoplasms remains controversial. In some studies, the carcinogenesis probability was higher in patients with MC than in those without MC (Wang et al., 2013; Zeng, 2010), whereas some studies proposed no statistical correlation of MC with colon cancer incidence (Biernacka-Wawrzonek et al., 2017). Given colon cancer incidence is high in the elderly populations and the people with constipation, the concomitant or causal relationship of MC with colon cancer is possible but remains to be clarified. In the present study, the patients with MC were older and colonic polyps were prone to be detected under MC background. The chronic malicious stimulation imposed by MC on the colonic mucosa was the reason for increased incidence of colon adenoma. Therefore, it is rational to postulate that MC is related to presence of colonic polyps and colon neoplasms. Nonetheless, a long-term follow-up of patients with MC is required to confirm the hypothesis.

The predilection sites of MC were referred to the distal colon or even the whole colon in some studies (Biernacka-Wawrzonek et al., 2017), or the left colon (Zapatier, Schneider & Parra, 2010). However, this was not analyzed further because of diverged colonoscopy report format from each medical center and the absence of clear documentation of lesion sites in most centers. This is a limitation of this study. It also hinted that medical staff generally ignored this specific disease and was not acute aware of the risk of concomitant diseases associated with MC. The heterogeneity of the data, diagnostic accuracy and study populations is another limitation of this study, although the multi-center design, centralized data procurement and large sample size may partially alleviate or overcome the limitation. Further, selection biases may be present in this study because of its retrospective nature and the urban setting of participating institutions. Finally, there might be a change in the rates of having colonoscopy in general population (e.g., colorectal cancer screening in asymptomatic subjects) in the past years, which in turn drove or linked to the increasing temporal-trend of MC detection rate. Taken together, caution should be used when interpreting or applying our findings to other populations.

## Conclusions

In China, the detection rate of MC has presented an incremental rising trend in the last 10 years. Regardless of gender, the detection rate of MC has increased with age. More females are affected than males, whereas the increase rate is higher in males than in females. Moreover, males in the elderly population are more prone to the MC detection rate. Colonic polyp is the most common concomitant disease of MC. Future studies are needed to elucidate the factors and causes associated with the increasing trends in MC detection rates.

##  Supplemental Information

10.7717/peerj.4483/supp-1 Supplemental Information 1 Raw data of 6,090 Chinese MC patients which was retrieved from data of 342,922 colonoscopy patients by engineer, Enming Zhou

## Additional Information and Declarations

### Data Availability

The following information was supplied regarding data availability: The raw data have been provided in a Supplemental File.

### Clinical Trial Registration

The following information was supplied regarding Clinical Trial registration: 301hnll-2017-05.
